# The effects of *Artemisia Sieberi*, *Achillea Fragrantissima*, and *Olea Europaea* leaves on the performance and physiological parameters in heat-stressed broiler chickens

**DOI:** 10.3389/fvets.2024.1410580

**Published:** 2024-06-17

**Authors:** Zeinab M. H. Mahasneh, Mohannad Abuajamieh, Anas Abdelqader, Mohmmad Al-Qaisi, Mohamed A. Abedal-Majed, Hosam Al-Tamimi, Hana Zakaria, Abdur-Rahman A. Al-Fataftah

**Affiliations:** ^1^Department of Animal Production, School of Agriculture, The University of Jordan, Amman, Jordan; ^2^Department of Animal Production, Faculty of Agriculture, Jordan University of Science and Technology, Irbid, Jordan

**Keywords:** antioxidant, broiler, health, heat stress, medicinal plants

## Abstract

High temperatures have detrimental effects on the performance and physiology of broiler chickens. Medicinal plants have various biological activities and may enhance the heat resistance of chickens during heat waves. Therefore, this study aimed to explore the potential roles of using specific local medicinal plants to alleviate the negative impacts of heat stress (HS) in broilers. In this study, 180 day-old chicks were used to investigate the effects of HS and dietary indigenous medicinal plants on growth performance, antioxidant biomarkers, and intestinal health. The chicks were assigned to six groups (18 pens with 10 chicks per pen) with three replicates each. In the first group, the chicks were kept under thermoneutral conditions (CON) and fed a basal diet. The other five groups were exposed to recurrent heat stress and fed a basal diet (T1, HS group) or supplemented with Artemisia Sieberi (1.25 g/kg of feed; T2), Achillea Fragrantissima (15 g/kg of feed; T3), *Olea europaea* (10 g/kg of feed; T4), and all the previous additives (all-in-one) combined at the same dose levels mentioned above (T5). At 21 days of age, the chicks from each group were exposed to two phases of heat stress: phase 1 from days 21 to 34 (34 ± 1°C) followed by phase 2 from days 35 to 39 (37 ± 1°C). The results indicate that HS significantly increased rectal temperature and respiration rate in broiler chickens. Feed intake and body weight gain were improved in all supplemented groups, while the feed conversion ratio was decreased in response to the dietary inclusion of medicinal plants. Additionally, glutathione peroxidase and immunoglobulin G levels were increased in the T3, T4, and T5 groups compared to the other groups. HS induced significant upregulated in the mRNA levels of heat shock protein 70 and interleukin-8, while the mRNA of occludin was decreased. The T3, T4, and T5 showed significantly decreased expression of hepatic HSP70 and ileum IL-8 genes and increased ileum mRNA occludin levels relative to the CON and T1 groups. In conclusion, supplementation with these plants enhances growth performance and maintains intestinal health sustaining the productivity of broiler chickens under HS conditions.

## 1 Introduction

Climate change is a pressing global challenge with far-reaching consequences on ecosystems, and it has profound implications for various sectors, including agriculture (1). Impact of climate change on farm animals is a growing concern as temperature and weather changes can affect their wellbeing, productivity, and performance (2). As the climate of the Earth changes, livestock farming is increasingly affected by rising temperatures, altered precipitation patterns, and more frequent extreme weather events. These environmental changes have direct and indirect impacts on farm animals (3, 4). Among the abundant challenges posed by climate change, heat stress (HS) is a critical factor in broiler chicken farming (5, 6). From HS to changes in forage availability and quality, farm animals are exposed to a variety of environmental stressors that can impact their performance and health (5, 7). Among farm animals, broiler chickens are especially vulnerable to environmental conditions. HS conditions notably affect broiler chicken performance and health (8, 9), leading to reduced growth rates, impaired feed efficiency (6, 7), and decreased productivity and economic returns for poultry farmers (10). Elevated temperatures can increase susceptibility to diseases by compromising the immune system in broiler chickens (11). Furthermore, HS disrupts the digestive process in broiler chickens, affecting nutrient absorption and feed digestibility by disrupting intestinal health. This is performed by promoting inflammation and damaging intestinal barriers (12, 13). HS can disrupt metabolic processes, leading to poor nutrient utilization in broiler chickens, resulting in weakened growth and performance. The gut microbiota is crucial for chicken health, and HS can cause imbalances, increasing the risk of gastrointestinal issues (14). To mitigate these adverse effects, various strategies can be employed, such as environmental modifications, breeding heat-tolerant breeds, and nutritional interventions (13, 15). Nutritional management is particularly important in alleviating HS effects on poultry (15, 16).

Medicinal plants are important substances in pharmaceutical products and dietary supplements because they contain high levels of bioactive substances such as polyphenols, flavonoids, and essential oils (17–20). These features indicate their remarkable antioxidant and anti-inflammatory properties. Jordan has rich flora expressed in a variety of plant species including medicinal plants. Many of these medicinal plants have been used as a self-medication for the treatment of dyslipidemia, diabetes, hypertension, cancer, and infertility (21). Despite some Jordanian medicinal plants having been used for various pharmaceutical purposes, their potential role in mitigating the negative effects of HS in broilers has not been explored. Broilers supplemented with medicinal plants have shown enhanced growth rates, improved feed conversion ratio, and better nutrient utilization (22). Additionally, these plants can boost immune function, reducing the risk of heat stress-induced infections (4, 23). The anti-inflammatory properties of medicinal plants also support gut health by maintaining the balance of gut microbiota (16).

*Artemisia Sieberi*, locally known as *Shieh*, is an annual herb belonging to the Asteraceae group. It typically grows to a height of 20–40 cm and is native to Jordan (21). This plant is known for its high concentrations of bioactive components, including phenolics, flavonoids, and steroids (21). Adding *Artemisia annua L*. to the diets of broiler chickens reared under HS significantly improved body weight, reduced oxidative stress biomarkers, and enhanced liver functions (24). Additionally, stressed broiler chickens (exposed to 34 ± 1°C for 8 h) provided with 1 g of *Artemisia annua* showed a decrease in plasma diamine oxidase levels, mRNA expression of heat shock protein 70 (HSP70), and interleukins in intestinal cells (25).

*Achillea Fragrantissima* (AFR), locally known as *Qaysoom*, is a widely used and highly valued medicinal herb in traditional Arabic medicine for preventing various diseases. The bioactive substances found in Achillea are phenolic acids and flavonoids (26). In a study by Eidrisha et al. (27), it was found that dietary inclusion of AFR (5 g/kg) significantly improved the body weight (BW), hepatic function, and feed conversion ratio (FCR) of quail birds under normal conditions. However, no significant effects on carcass traits were observed. Further research is needed to investigate the use of AFR in poultry feed, especially under HS conditions. Olive (*Olea europaea*) is a medicinal herb that is rich in polyphenols such as pinoresinol, hydroxytyrosol, oleuropein, and tyrosol (28), which have powerful antibacterial, antioxidant, and anti-inflammatory effects. Agah et al. (29) found no significant effects of olive leaves on the performance parameters of heat-stressed broilers (33°C). Olive leaves have been discovered to lower lipid profiles, improve liver functions, and increase antioxidant enzymes such as glutathione peroxidase. These findings have prompted researchers to investigate the potential of these medicinal plants from Jordan in alleviating the adverse effects of heat stress in poultry, due to their antioxidant properties. Therefore, the study was designed to assess the antioxidant properties of indigenous medicinal plants (*Artemisia Sieberi*, *Achillea Fragrantissima*, and *Olea Europaea leaves*) on the growth performance, intestinal morphology, and health of heat-stressed broiler chickens.

## 2 Materials and methods

### 2.1 Ethical approval

The Animal Ethics Committee of the Deanship of Scientific Research at the University of Jordan (Amman, Jordan) approved the experimental procedures used in this study.

### 2.2 Plant collection and phytochemical analysis

Medicinal plants, including *Artemisia sieberi* and *Achillea fragrantissima*, were collected from the Badia region in Al Safawi, and *Olea europaea* leaves were collected from Jerash City in Kufr Khall. The plant species were taxonomically identified by Prof. Mahfouz Abu-Zanat from the Department of Animal Production at the School of Agriculture, The University of Jordan. Voucher specimens (#22/11/88, #16/7/88, and #23/1/21, respectively) were deposited at the herbarium of the Department of Biology at the School of Science, The University of Jordan. According to reference (30), the total phenolic and flavonoid content, 2,2-diphenyl-1-picrylhydrazyl (DPPH) radical scavenging activity, and ferric ion reducing antioxidant potential (FRAP) of the experimental plants were determined (see Table 1). The chemical constituents of the oil obtained by hydro-distillation were analyzed using gas chromatography-mass spectrometry (see Table 2).

**Table 1:** Phytochemical evaluations of aqueous extracts of *Artemisia Sieberi*, *Achillea Fragrantissima*, and *Olea Europea* were used in this research.

Medicinal plants extracts	Phytochemical evaluations			
	Phenolic (mgQE/100 mg)	Flavonoids (mgQE/100 mg)	DPPH (ppm)	FRAP (%)
*Artemisia Sieberi*	250	23.04	17.53	15.17
*Achillea Fragrantissima*	291.66	30.23	33.15	10.11
*Olea Europea*	483.33	27.00	19.57	21.81

**Table 2:** Composition of medicinal plants essential oil.

*Achillea Fragrantissima*		*Artemisia Sieberi*		*Olea Europea leaves*	
KI^a^	Rt^b^	KI^a^	Rt^b^	KI^a^	Rt^b^
906	6.584	908	7.706	922	9.033
922	9.035	914	8.274	924	9.274
929	9.798	921	8.99	927	9.547
998	11	924	9.265	999	11.178
1,001	11.338	996	10.679	1,001	11.371
1,008	12.342	998	11.003	1,004	11.822
1,011	12.856	1,000	11.294	1,008	12.365
1,090	14.764	1,006	12.04	1,088	14.367
1,092	15.052	1,007	12.233	1,089	14.655
1,097	16.037	1,008	12.347	1,099	16.448
1,099	16.408	1,010	12.689	1,110	18.449
1,106	17.699	1,012	12.946	1,188	19.89
1,107	17.811	1,088	14.342	1,195	21.426
1,182	18.588	1,089	14.487	1,199	22.419
1,199	22.452	1,090	14.697	1,285	25.108
1,285	25.135	1,091	14.958	1,290	26.358
1,286	25.377	1,093	15.217	1,374	27.75
1,291	26.774	1,093	15.33	1,376	28.264
1,292	27.067	1,097	15.997	1,378	29
1,401	29.407	1,098	16.142	1,381	29.736
1,384	30.688	1,099	16.32	1,384	30.759
1,468	31.287	1,100	16.594	1,469	31.56
1,471	32.327	1,102	17.047	1,476	33.842
1,568	36.375	1,104	17.371	1,477	34.386
1,571	37.5	1,105	17.533	1,479	34.855

a KI, Kovats’ indices.

b RT, retention time.

### 2.3 Animals and experimental design

A total of 180 1 day-old Ross 308 broiler chicks were obtained from a local commercial hatchery and placed in brooders with dimensions of 188 cm × 82 cm × 68 cm, featuring a wire mesh floor. The birds were reared under optimal ambient temperature conditions and provided with *ad libitum* access to feed (Table 3) and water. At 1 day of age, the chicks were randomly allocated into six dietary treatments, each with three replicates (consisting of 10 birds per replicate), and were supplemented with feed additives.

**Table 3:** Ingredients and nutrient composition of the diets fed during the experiment.

Ingredients	Unit	Starter	Grower	Finisher
Corn	kg	581.00	626.00	662.00
Soybean meal	kg	375.00	325.00	280.00
Soy oil	kg	5.00	12.00	22.00
CaCo3	kg	14.00	12.00	11.00
Premix	kg	25.00	0.00	0.00
Calculated analysis*				
Weight	kg	1000.00	1000.00	1000.00
Dry matter	%	88.00	88.00	88.00
Metabolizable energy	Kcal	3000.00	3100.00	3200.00
Crude protein	%	22.40	20.50	18.70
Calcium	%	0.96	0.87	0.79
Avi. phosphorus	%	0.48	0.44	0.40
Dig. Lysine	%	1.32	1.18	1.08
Dig. Methionine + Cystine	%	1.0	0.92	0.86
Dig. Threonine	%	0.88	0.79	0.72
Dig. Valine	%	1.00	0.91	0.84
Sodium	%	0.16	0.16	0.16
Chloride	%	0.20	0.20	02.0

At 21 days of age, treatments 2 to 6 were subjected to intermittent heat stress (phase 1) in environmentally controlled chambers. The temperature during phase 1 was maintained at 34 ± 1°C with a relative humidity of 49 ± 1% for 15 consecutive days (from days 21 to 34) for 4 h per day from 11:00 to 15:00. This was followed by phase 2, where the temperature was increased to 37 ± 1°C with a relative humidity of 52 ± 1% for 5 consecutive days (from days 35 to 39) for 4 h per day from 11:00 to 15:00.

The treatments were distributed as follows:

1. CON: Thermoneutral control group fed a basal diet.
2. T1: Heat-stressed control group fed a basal diet.
3. T2: Heat-stressed group supplemented with *Artemisia Sieberi* at a rate of 1.25 g/kg of feed.
4. T3: Heat-stressed group supplemented with *Achillea Fragrantissima* at a rate of 15 g/kg of feed.
5. T4: Heat-stressed group supplemented with *Olea europaea* at a rate of 10 g/kg of feed.
6. T5: Heat-stressed group supplemented with all previous additives combined at the same dose levels mentioned above.

### 2.4 Environmental parameters

Ambient temperature (Ta) and relative humidity (RH%) were recorded at hourly intervals throughout the entire study using thermo-loggers located within the laboratory at the birds’ level. Mean Ta (temperature) and RH% (relative humidity) for each day were calculated from these records (Ta = 28 ± 1°C; RH% = 46% ± 1), phase 1: (Ta = 34 ± 1°C; RH% = 49 ± 1) and phase 2: (Ta = 37 ± 1°C, RH% = 52 ± 1).

Broiler chicks (180) were divided into six groups. The control group was fed a basal diet and kept at environmental condition (28 ± 1°C; RH% = 46% ± 1), while the second group (T1, HS group) was fed a basal diet and at 21 days of age was exposed to cyclic HS (temperature = 34 ± 1°C with a relative humidity of 49 ± 1%) for 4 h/day (from days 21 to 34) from 11:00 to 15:00. This was followed by phase 2, where the temperature was increased to 37 ± 1°C with a relative humidity of 52 ± 1% for 5 consecutive days (from days 35 to 39) for 4 h per day from 11:00 to 15:00. The treated groups (T2, T3, T4, and T5) were fed basal diet supplemented with Artemisia Sieberi, Achillea Fragrantissima, Olea europaea, and their combinations at a rate of 1.25, 15, 10, 26.25 g/kg respectively.

### 2.5 Performance parameters

Weekly feed intake (FI) and body weights (BW) were measured on days 0, 7, 14, 21, 28, 35, and 39 (the final day of the experiment). Body weight gain (BWG) was determined by subtracting the initial weight of each bird from its weight at the end of the study. The feed conversion ratio (FCR) was calculated weekly during the experiment using the formula: FCR = (average feed consumed in grams per interval/average BWG in grams during the same interval). Daily mortalities were noted for each replicate in the treatment, and the FCR was adjusted accordingly.

### 2.6 Carcass parameters

In total, nine birds per treatment were randomly selected, weighed, and fasted overnight. The birds were euthanized and immediately processed to collect intestinal samples and organs required for analysis. The carcass weight (g), dressing percentage (%), liver, heart, and spleen were weighed and adjusted as percentages of the live body weight.

### 2.7 Physiological responses

**Figure 1:**
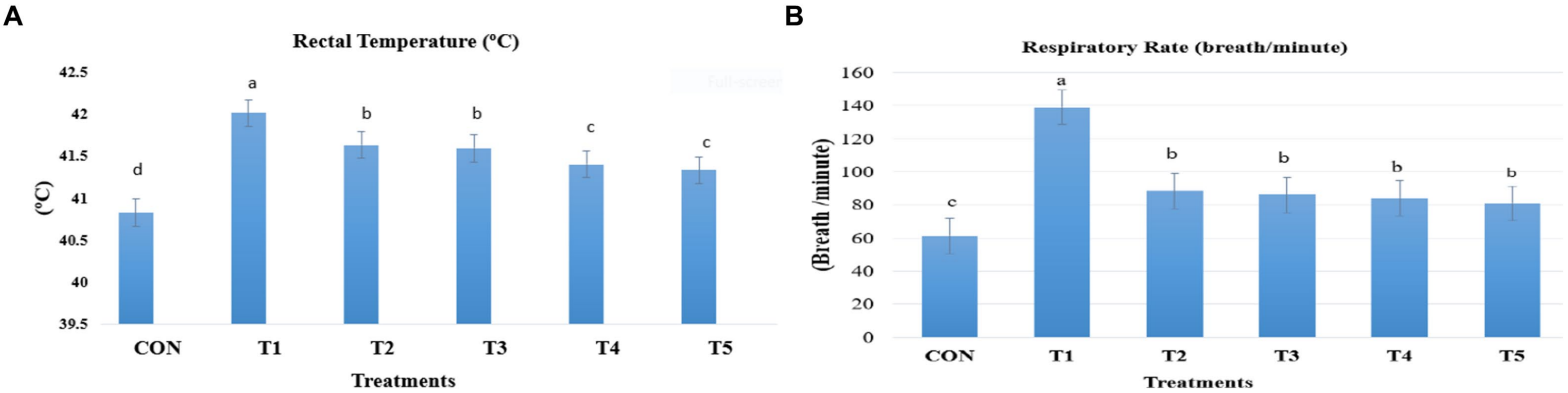
Change in rectal temperatures **(A)** and respiration rates **(B)** of broiler as a response to dietary feed additives supplementation. Broiler chickens (Ross308) were fed a basal diet (CON; Ta = 28 ± 1°C; RH% = 46% ± 1), while the other chicken groups were exposed to intermittent heat stress phase 1 (Ta = 34 ± 1°C, RH% = 49 ± 1) and phase 2 (Ta = 37 ± 1°C, RH% = 52 ± 1) after which thermoneutrality was resumed (Ta = 28 ± 1°C; RH% = 46% ± 1), and fed diets without supplementation (T1, heat stress group), or fed with 1.25 g of *Artemisia Sieberi*/kg (T2), 15 g of *Achillea Fragrantissima*/kg diet (T3), 10 g of *Olea europaea*/kg diet (T4) and finally T5 supplemented with all previous additives combined at the same dose levels mentioned above. ^2^Standard error of the mean. ^a–c^ Means with different superscripts within the same row differ significantly (*p* < 0.05), according to the Tukey test.

Rectal temperature was recorded twice daily for nine birds per treatment during the heat exposure period (21–39 days of age) using portable digital thermometers (GLM700) connected to a very fine probe. The probe was inserted a maximum of 4–5 cm inside the vent of each bird. The respiratory rate was measured by counting the panting breaths of the birds for 15 s, and this value was multiplied by 4 to determine the number of respiratory breaths per minute. Nine birds per treatment were used to measure the respiratory rate (See Figure 1).

### 2.8 Blood parameters

A total of five birds/treatments were randomly selected for blood samples to measure cortisol, glutathione peroxidase (GSH-Px), and immunoglobulin G (IgG). Blood was drawn from the jugular vein of each bird into heparinized tubes and immediately centrifuged at 3500 rpm for 15 min to obtain plasma. The blood samples were then stored frozen at −20°C for later analysis. The blood kits were obtained from Biotechnology Co., Ltd., in Wuhan City, China. Plasma cortisol concentrations were assessed using commercial kits (Cortisol ELISA Kit, ELK Biotechnology, Wuhan, China). The levels of GSH-Px were assessed by commercially available kits according to the manufacturer’s instructions. Plasma immunoglobulin levels (IgG) were measured on flat-bottom 96-well plates using chicken IgG enzyme-linked immunosorbent assay (ELISA) quantification kits (Biotechnology, Wuhan, China) according to the manufacturer’s instructions.

### 2.9 Intestinal parameters

Duodenum, jejunum, and ileum samples were collected from a total of 54 birds (9 birds/treatment) at 39 days of age and were fixed in 10% buffered formalin for histological analysis as described in reference (31).

### 2.10 Gene expression (hepatic and ileum samples)

RNA and mRNA abundance were extracted following the previously described method (32). A section of hepatic and ileal tissues (40 mg each) was collected from each of the 36 birds (6 birds per treatment at 39 days of age) and stored in a DNA/RNA shield (Zymo, Irvine, CA, United States) to preserve RNA integrity. Total RNA was extracted from the samples and then converted to complementary DNA (cDNA) for quantitative PCR analysis. Quantitative RT-PCR (QRT-PCR) (Qiagen, Hilden, Germany) was used to determine the mRNA levels in hepatic and ileum samples. The relative mRNA abundance for each gene was normalized to the GAPDH expression as a housekeeping gene. Primers were designed for the constitutively expressed mRNAs (Table 4). Gene expression levels were analyzed using the 2^−ΔΔCT^ method (33). The data were normalized to the average mRNA abundance in the control samples (set as a reference value of 1) and expressed as fold change relative to the control.

**Table 4:** Primers used in the current investigation.

Gene name	Accession number	Primers
GAPDH	NM_204305	F-CTGGCAAAGTCCAAGTGGTG R-AGCACCACCCTTCAGATGAG
IL-8	AJ009800	F-GCCCTCCTCCTGGTTTCAG R-TGGCACCGCAGCTCATT
OCLN	XM_025144248	F-ACGGCAGCACCTACCTCAA R-GGGCGAAGAAGCAGATGAG
HSP70	NM_0010066851	F-AGCGTAACACCACCATTCC R-TGGCTCCCACCCTATCTC

Heat Shock Protein 70 (HSP70), Occludin (OCLN), Interleukin-8 (IL-8), and glyceraldehyde-3-phosphate (GAPDH). F, forward; R, reverse.

### 2.11 Statistical analysis

The chicks were randomly assigned to six treatments in a completely randomized design with three replicate pens per treatment. Each treatment had 30 birds, with 10 birds in each replicate pen. The replicate pen was considered the experimental unit for performance variables. Individual birds were considered the experimental unit for the analysis of blood samples, intestinal tissues, and gene expression. The effect of treatment on variables was analyzed using PROC MIXED in SAS 9.4. The results were presented as least squares means, and significance was determined at a *p*-value of <0.05, with a tendency for the difference at a *p*-value of <0.10. The means were separated using Tukey’s multiple range test.

## 3 Result

### 3.1 Growth indices and feed intake

During weeks 3, 5, and 6, there was no significant difference in FI between the CON treatment and T1 treatment (*p* > 0.05; Table 5). FI was decreased during weeks 1 and 2 (11 and 14%, respectively, *p* ≤ 0.05; Table 5) in the T5 and T2 treatments compared to the T1 treatment. Similarly, FI was lower in the T5 treatment than the CON treatment (28%, *p* = 0.02; Table 5) during week 4. During weeks 1, 2, 3, and 5, BWG did not differ between CON treatment and T1 treatments (*p* > 0.05; Table 5). However, the BWG of the broiler was increased during week 4 (29%, *p* = 0.04; Table 6) in the T2 treatment relative to the broiler in stressed groups without supplementation. BWG (from days 35 to 39) was decreased in HS treatments compared with CON treatment (*p* = 0.01; Table 5). Regarding FCR, it did not differ between CON treatment and HS treatments during weeks 1 and 3 of the experiment period (*p* > 0.05; Table 5). However, during week 2, FCR was decreased in T1 (heat stress) treatment compared to CON treatment. FCR was increased significantly during weeks 3–6 (29%, *p* < 0.05; Table 5) in HS treatment (T1) compared to CON treatment. Moreover, the FCR of the treated broiler with the medicinal plant (T2, T3, T4, and T5) was improved compared to the HS (T1) treatment (*p* < 0.05; Table 5).

**Table 5:** Mean feed intake, body weight gain (BWG), and feed conversion ratio (FCR) of broiler chickens treated with different types of medicinal plants during heat stress conditions.

Item	Treatment^a^						SEM^b^	*p*-value
	CON	T1	T2	T3	T4	T5		
Feed intake (g/bird)								
Week1	170.00^ab^	175.00^a^	163.67^ab^	161.33^ab^	161.33^ab^	155.67^b^	3.87	0.05
Week2	398.00^ab^	422.33^a^	393.67^ab^	359.67^b^	401.00^ab^	389.00^ab^	9.98	0.02
Week3	851.93	877.67	856.33	901.22	917.52	867.96	32.31	0.67
Week4	1123.33^a^	1016.67^ab^	1023.33^ab^	1011.67^ab^	1010.00^ab^	805.00^b^	51.06	0.02
Week5	465.00	520.00	518.33	573.33	555.00	476.67	55.63	0.72
Week6	538.33	463.33	453.33	410.00	366.67	421.67	37.01	0.09
Body Weight Gain (g/bird)								
Week1	127.67	132.33	124.33	124.33	125.00	122.33	4.78	0.74
Week2	341.00	306.00	329.67	315.00	311.67	317.33	9.72	0.19
Week3	514.67	448.00	467.67	482.00	533.33	506.67	25.76	0.25
Week4	609.24^ab^	510.33^b^	662.81^a^	592.63^ab^	604.70^ab^	606.24^ab^	26.63	0.04
Week5	305.00	263.00	269.52	299.37	420.97	276.43	38.79	0.11
Week6	318.33^a^	215.00^b^	231.67^ab^	210.00^b^	190.00^b^	218.33^b^	20.31	0.01
Feed Conversion Ratio (g feed/g gain)								
Week1	1.33	1.32	1.31	1.30	1.29	1.27	0.03	0.72
Week2	1.67^b^	1.38^a^	1.19^b^	1.14^b^	1.28^ab^	1.22^ab^	0.03	0.01
Week3	1.65	1.96	1.84	1.86	1.73	1.72	0.07	0.06
Week4	1.32^d^	1.99^a^	1.54^cd^	1.71^abc^	1.67^bc^	1.86^ab^	0.07	0.01
Week5	1.53^ab^	1.97^a^	1.92^ab^	1.91^ab^	1.39^b^	1.73^ab^	0.12	0.03
Week6	1.69^c^	2.15^a^	1.95^b^	1.95^b^	1.92^b^	1.93^b^	0.03	0.00

a Broiler chickens (Ross308) were fed a basal diet (CON; Ta = 28 ± 1°C; RH% = 46% ± 1), while the other chicken groups were exposed to intermittent heat stress phase 1 (Ta = 34 ± 1°C, RH% = 49 ± 1) and phase 2 (Ta = 37 ± 1°C, RH% = 52 ± 1) after which thermoneutrality was resumed (Ta = 28 ± 1°C; RH% = 46% ± 1), and fed diets without supplementation (T1, heat stress group), or fed with 1.25 g of *Artemisia Sieberi*/kg (T2), 15 g of *Achillea Fragrantissima*/kg diet (T3), 10 g of *Olea europaea*/kg diet (T4) and finally T5 supplemented with all previous additives combined at the same dose levels mentioned above.

b Standard error of the mean.^a–b^Means with different superscripts within the same row differ significantly (*p* < 0.05), according to the Tukey test.

**Table 6:** Changes in carcass characteristics of broiler chickens in response to dietary supplementation of various types of medicinal plants during heat stress conditions.

Variables	Treatment^a^						SEM^b^	*p*-value
	CON	T1	T2	T3	T4	T5		
Live weight (g)	2782.22	2732.22	2801.11	3063.33	2748.75	2888.89	118.87	0.38
Carcass weight (g)	1858.99	1795.40	1837.08	1996.27	1844.06	1892.69	71.89	0.40
Dressing, %	68.98	65.59	65.83	65.68	67.06	68.40	1.75	0.59
Liver weight (g)	59.06	62.62	68.82	70.01	56.50	68.26	3.93	0.07
Heart weight (g)	13.53	14.56	14.23	16.74	15.35	14.68	0.85	0.17
Spleen weight (g)	2.48b	2.20	2.43	3.70	3.56	3.00	0.27	0.17
Liver, % of BW	3.25	3.48	3.76	3.47	3.24	3.79	0.19	0.10
Heart, % of BW	0.73	0.81	0.78	0.83	0.83	0.77	0.04	0.57
Spleen, % of BW	0.13^ab^	0.12^b^	0.13^ab^	0.18^a^	0.19^a^	0.16^ab^	0.01	<0.05

a Broiler chickens (Ross308) were fed a basal diet (CON; Ta = 28 ± 1°C; RH% = 46% ± 1), while the other chicken groups were exposed to intermittent heat stress phase 1 (Ta = 34 ± 1°C, RH% = 49 ± 1) and phase 2 (Ta = 37 ± 1°C, RH% = 52 ± 1) after which thermoneutrality was resumed (Ta = 28 ± 1°C; RH% = 46% ± 1), and fed diets without supplementation (T1, heat stress group), or fed with 1.25 g of *Artemisia Sieberi*/kg (T2), 15 g of *Achillea Fragrantissima*/kg diet (T3), 10 g of *Olea europaea*/kg diet (T4) and finally T5 supplemented with all previous additives combined at the same dose levels mentioned above.

b Standard error of the mean.^a–b^Means with different superscripts within the same row differ significantly (*p* < 0.05), according to the Tukey test.

### 3.2 Carcass characteristics

On day 39, carcass traits such as carcass weight (g), dressing percentage, heart weight (g), liver weight (g), spleen weight (g), heart percentage, and liver percentage did not show significant differences between the CON treatment and HS (T1) treatments (*p* > 0.05; Table 6). Spleen percentage was higher in the T3 and T4 treatments (50 and 58%, respectively, *p* < 0.05; Table 6) than the untreated heat-stressed group (T2).

### 3.3 Physiological responses

During the intermittent HS (Ta = 34.50 ± 0.05°C, RH% = 49.23 ± 0.21), both rectal temperature and respiration rate were significantly increased in the HS treatment (T1) compared to the other groups (*p* < 0.05; Figure 1A). In contrast, all supplemented medicinal plants significantly decreased the rectal temperature and respiration rates (Figure 1B) of the broiler. Rectal temperature was lower in the broiler given diets T4 and T5 treatments than the T2 and T3 treatments (*p* < 0.05). All-supplemented feed additives reduced respiration rates, but these values are still above the values of the CON group (*p* < 0.05).

### 3.4 Blood parameters

On day 39, serum cortisol levels did not differ between the CON and T1 treatments (*p* > 0.05; Table 7). However, immunoglobulin G levels were increased by 0.55% (*p* < 0.05; Table 7) in the T4 treatment compared to the T1 treatment (heat-stressed group). Circulating glutathione peroxidase levels were increased significantly (*p* < 0.05; Table 7) in the T5 and T3 groups compared to the stressed group of broilers.

**Table 7:** Changes in serum cortisol, immunoglobulins G, and glutathione peroxidase of broiler chickens in response to dietary supplementation of various types of medicinal plants during heat stress conditions.

Parameters	Treatment^a^						SEM^b^	*p*-value
	CON	T1	T2	T3	T4	T5		
Cortisol, ng/ml	27.36	25.47	43.07	13.32	21.35	20.18	12.04	0.63
Immunoglobulin G, ng/ml	7.74^ab^	5.53^b^	7.18^ab^	8.08^ab^	12.40^a^	7.24^ab^	1.40	0.04
Glutathione peroxidase, ng/ml	88.14^ab^	53.46^b^	80.05^ab^	113.49^a^	70.66^ab^	112.76^a^	12.97	0.02

a Broiler chickens (Ross308) were fed a basal diet (CON; Ta = 28 ± 1°C; RH% = 46% ± 1), while the other chicken groups were exposed to intermittent heat stress phase 1 (Ta = 34 ± 1°C, RH% = 49 ± 1) and phase 2 (Ta = 37 ± 1°C, RH% = 52 ± 1) after which thermoneutrality was resumed (Ta = 28 ± 1°C; RH% = 46% ± 1), and fed diets without supplementation (T1, heat stress group), or fed with 1.25 g of *Artemisia Sieberi*/kg (T2), 15 g of *Achillea Fragrantissima*/kg diet (T3), 10 g of *Olea europaea*/kg diet (T4) and finally T5 supplemented with all previous additives combined at the same dose levels mentioned above.

b Standard error of the mean.^a–b^Means with different superscripts within the same row differ significantly (*p* < 0.05), according to the Tukey test.

### 3.5 Intestinal parameters

**Figure 2:**
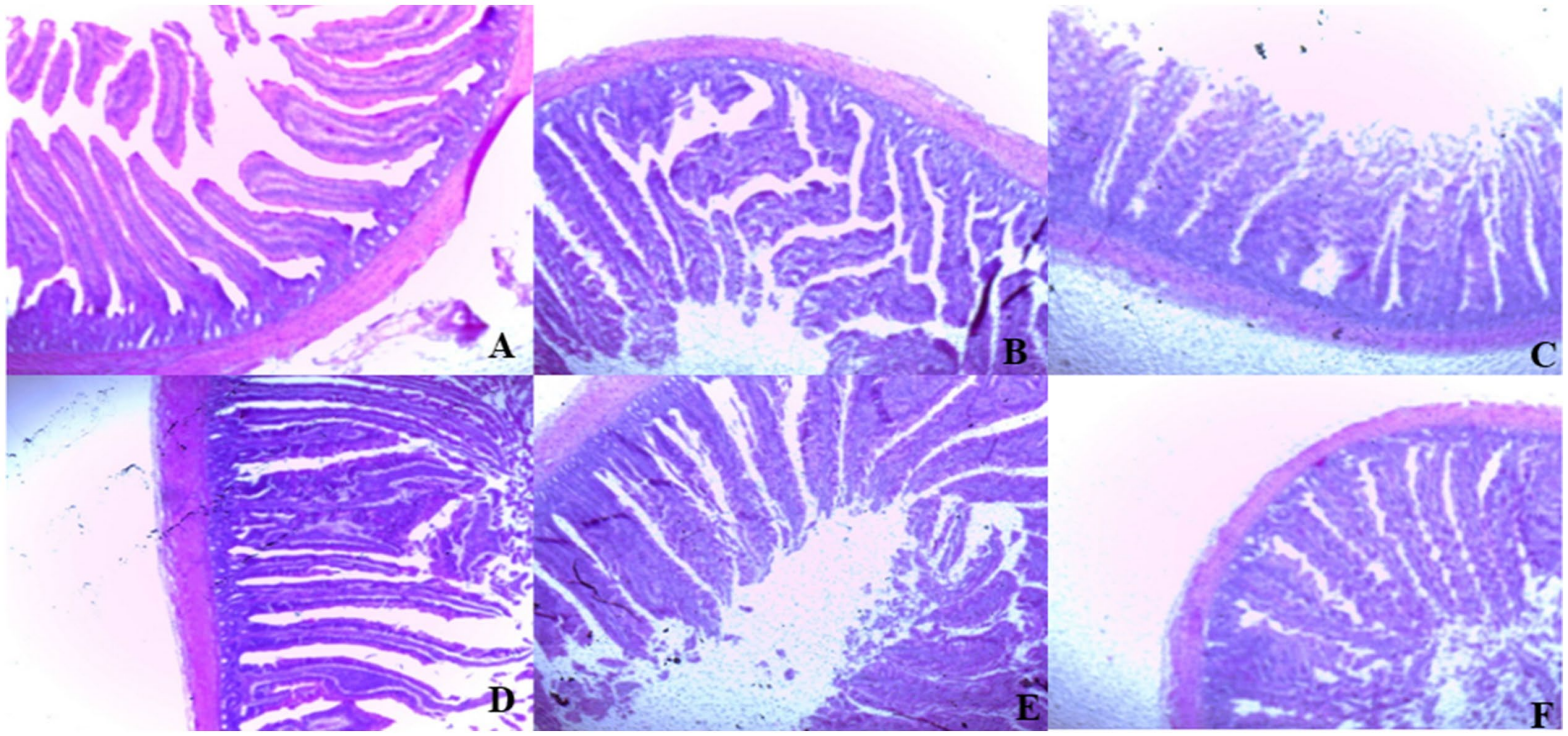
**(A–F)** Changes of duodenum morphology in all experimental groups. Broiler chickens were fed a basal diet (**A**; CON; Ta = 28 ± 1°C; RH% = 46% ± 1), while the other chicken groups were exposed to intermittent heat stress phase 1 (Ta = 34 ± 1°C, RH% = 49 ± 1) and phase 2 (Ta = 37 ± 1°C, RH% = 52 ± 1) after which thermoneutrality was resumed (Ta = 28 ± 1°C; RH% = 46% ± 1), and fed diets without supplementation (**B**; T1, heat stress group), or fed with 1.25 g of *Artemisia Sieberi*/kg diet (T2; **C**), 15 g of *Achillea Fragrantissima*/kg diet (T3; **D**), 10 g of *Olea europaea*/kg diet (T4; **E**) and finally T5 **(F)** supplemented with all previous additives combined at the same dose levels mentioned above. ^2^Standard error of the mean. ^a–b^ Means with different superscripts within the same row differ significantly (*p* < 0.05), according to the Tukey test.

**Figure 3:**
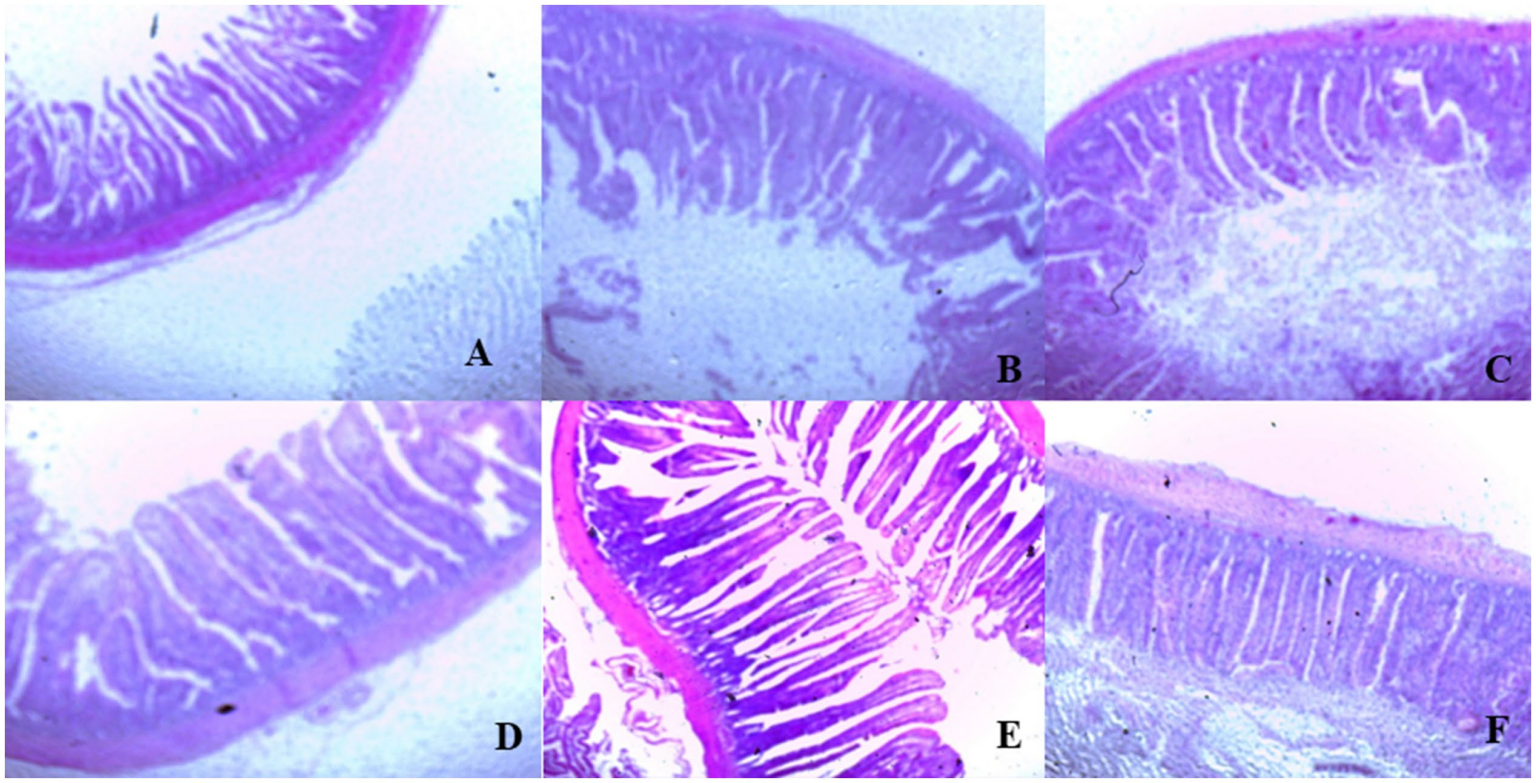
**(A–F)** Changes of jejunum morphology in all experimental groups. Broiler chickens were fed a basal diet (**A**; CON; Ta = 28 ± 1°C; RH% = 46% ± 1), while the other chicken groups were exposed to intermittent heat stress phase 1 (Ta = 34 ± 1°C, RH% = 49 ± 1) and phase 2 (Ta = 37 ± 1°C, RH% = 52 ± 1) after which thermoneutrality was resumed (Ta = 28 ± 1°C; RH% = 46% ± 1), and fed diets without supplementation (**B**; T1, heat stress group), or fed with 1.25 g of *Artemisia Sieberi*/kg diet (T2; **C**), 15 g of *Achillea Fragrantissima*/kg diet (T3; **D**), 10 g of *Olea europaea*/kg diet (T4; **E**) and finally T5 **(F)** supplemented with all previous additives combined at the same dose levels mentioned above.

**Figure 4:**
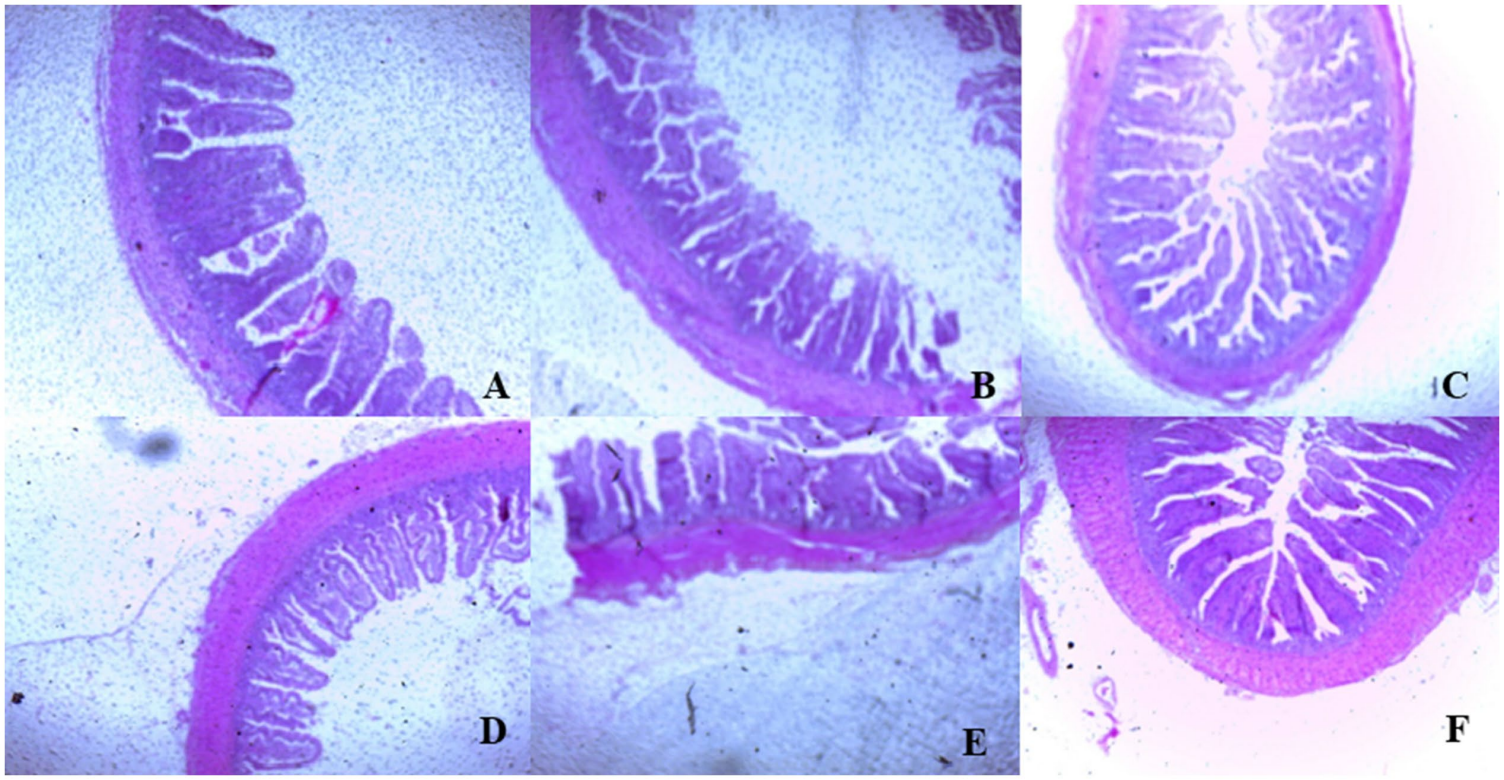
**(A–F)** Changes of ileum morphology in all experimental groups. Broiler chickens were fed a basal diet (**A**; CON; Ta = 28 ± 1°C; RH% = 46% ± 1), while the other chicken groups were exposed to intermittent heat stress phase 1 (Ta = 34 ± 1°C, RH% = 49 ± 1) and phase 2 (Ta = 37 ± 1°C, RH% = 52 ± 1) after which thermoneutrality was resumed (Ta = 28 ± 1°C; RH% = 46% ± 1), and fed diets without supplementation (**B**; T1, heat stress group), or fed with 1.25 g of *Artemisia Sieberi*/kg diet (T2; **C**), 15 g of *Achillea Fragrantissima*/kg diet (T3; **D**), 10 g of *Olea europaea*/kg diet (T4; **E**) and finally T5 **(F)** supplemented with all previous additives combined at the same dose levels mentioned above.

On day 39, T3 treatment significantly increased villus height in the duodenum compared to T1 treatment (*p* < 0.01; Table 8). In the jejunum, both T4 and T3 treatments led to higher villus height than the stressed group (T1; *p* < 0.01). However, in the ileum, villus height was decreased in the T4 and T5 treatments compared to the T1 treatment (*p* < 0.01; Table 8). Additionally, T2 treatment increased villus depth in the duodenum compared to T1 treatment (*p* < 0.01). In the jejunum, villus height was decreased in the T4 and T1 treatment (*p* < 0.01), while in the ileum, villus depth was decreased in the T4 treatment compared to CON (*p* < 0.01). Furthermore, T3 and T4 treatments increased villus surface area in the duodenum compared to T1 treatment (*p* < 0.01; Table 8). In the ileum, T4 treatment resulted in increased villus surface area compared to T1 treatment (*p* < 0.01). Figures 2–4 illustrate the detrimental effects of HS on villi structure in T1 compared to CON duodenum, jejunum, and ileum. However, medicinal plant treatments showed minimal damage to the villi tips compared to T1 treatment.

**Table 8:** Changes in intestinal morphology of broiler chickens in response to dietary supplementation of various types of medicinal plants during heat stress conditions.

Parameters	Treatment^a^						SEM^b^	*p*-value
	CON	T1	T2	T3	T4	T5		
Duodenum								
Villi Height, μm	1180^abc^	990^d^	1150^bcd^	1,350^a^	1000^cd^	1230^ab^	40	<0.05
Crypt Depth, μm	150^b^	210^a^	180^a^	190^ab^	187^ab^	160^ab^	7	<0.05
Villus Surface Area, μm	593^a^	497^b^	433^ab^	678^a^	471^a^	811^ab^	40	<0.05
Jejunum								
Villi Height, μm	800^ab^	530^c^	600^bc^	920^a^	830^a^	720^abc^	60	<0.05
Crypt Depth, μm	120^b^	160^a^	140^ab^	160^a^	120^b^	140^ab^	60	<0.05
Villus Surface Area, μm	352^ab^	249^b^	283^ab^	433^a^	312^ab^	339^ab^	40	0.02
Ileum								
Villi Height, μm	720^a^	540^b^	660^ab^	550^b^	740^a^	750^a^	30	<0.05
Crypt Depth, μm	120^b^	160^a^	130^ab^	140^ab^	120^b^	140^ab^	7	<0.05
Villus Surface Area, μm	271^a^	288^b^	31^ab^	276^ab^	302^a^	283^a^	40	<0.05

a Broiler chickens (Ross308) were fed a basal diet (CON; Ta = 28 ± 1°C; RH% = 46% ± 1), while the other chicken groups were exposed to intermittent heat stress phase 1 (Ta = 34 ± 1°C, RH% = 49 ± 1) and phase 2 (Ta = 37 ± 1°C, RH% = 52 ± 1) after which thermoneutrality was resumed (Ta = 28 ± 1°C; RH% = 46% ± 1), and fed diets without supplementation (T1, heat stress group), or fed with 1.25 g of *Artemisia Sieberi*/kg diet (T2), 15 g of *Achillea Fragrantissima*/kg diet (T3), 10 g of *Olea europaea*/kg diet (T4) and finally T5 supplemented with all previous additives combined at the same dose levels mentioned above.

b Standard error of the mean.^a–b^Means with different superscripts within the same row differ significantly (*p* < 0.05), according to the Tukey test.

### 3.6 Gene expression

**Figure 5:**
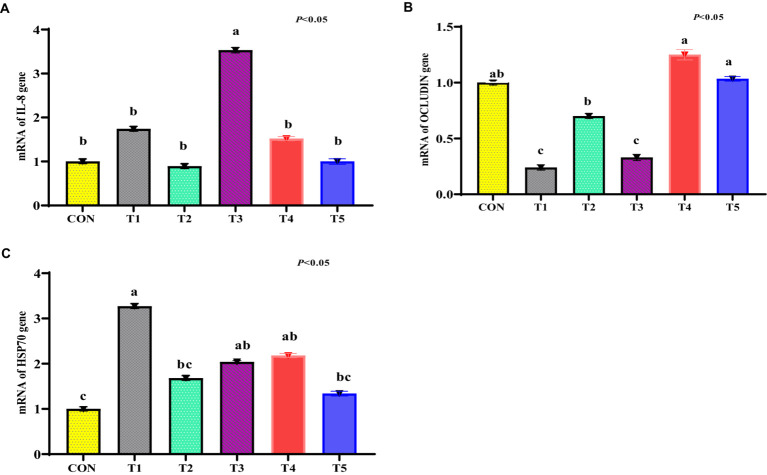
**(A–C)** Expression of interleukin-8 (IL-8; **A**), occludin (OCLN; **B**), and HSP70 **(C)** genes in the ileum tissue of heat-challenged broiler chickens treated with dietary various medicinal plants. Broiler chickens (Ross308) were fed a basal diet (CON; Ta = 28 ± 1°C; RH% = 46% ± 1), while the other chicken groups were exposed to intermittent heat stress phase 1 (Ta = 34 ± 1°C, RH% = 49 ± 1) and phase 2 (Ta = 37 ± 1°C, RH% = 52 ± 1) after which thermoneutrality was resumed (Ta = 28 ± 1°C; RH% = 46% ± 1), and fed diets without supplementation (T1, heat stress group), or fed with 1.25 g of *Artemisia Sieberi*/kg diet (T2), 15 g of *Achillea Fragrantissima*/kg diet (T3), 10 g of *Olea europaea*/kg diet (T4) and finally T5 supplemented with all previous additives combined at the same dose levels mentioned above. ^2^Standard error of the mean. ^a–b^ Means with different superscripts within the same row differ significantly (*p* < 0.05), according to the Tukey test.

The mRNA abundance for *IL-8* was increased in the ileum tissue of the stressed broiler (T1 group) compared to other treatments (*p* < 0.05; Figure 5A). There were no significant differences in mRNA levels of *IL-8* in the T2, T3, T4, and T5 treatments relative to the CON treatment (*p* > 0.05). The mRNA abundance for the *OCLN* gene tended to be significant in the ileum tissue of the stressed treatment (T1 group) compared to the CON treatment (*p* > 0.05; Figure 5B). The mRNA abundance for *OCLN* in the ileum tissue of the T5 and T4 treatments was like the CON treatment (*p* < 0.05; Figure 5B). The mRNA abundance for *HSP70* tended to increase in the hepatic tissue of the T1 group compared to the CON treatment (*p* < 0.05; Figure 5C). The mRNA abundance for *HSP70* tended to be decreased in the hepatic tissue of the T2 and T4 treatments compared to the T1 treatment (*p* < 0.05; Figure 5C). There were no significant differences in the mRNA RNA in the hepatic tissue of the T3 and T4 treatments relative to the T1 treatment (*p* > 0.05; Figure 5C).

## 4 Discussion

This study aimed to assess the effects of dietary supplementation with medicinal plants on broiler chickens during intermittent HS. Performance parameters such as BWG, FI, FCR, carcass characteristics, rectal temperature, respiratory rate, blood parameters (cortisol, immunoglobulins, and glutathione peroxidase), expression of *HSP70*, *IL-8*, and *OCLN* genes, and intestinal histology were evaluated. Several experiments have investigated the impact of high environmental temperatures on the performance and health of broilers. In this experiment, broiler chickens treated with medicinal plants showed slightly increased FI (except for the mixture of herbs groups; T5) compared to the stressed treatment. They also exhibited increased BWG when treated with *Artemisia Sieberi* and improved FCR in all medicinal plant treatments compared to the CON/HS treatment. These performance results are consistent with other studies (4, 15, 27, 34–36) that have reported improvements in FI, BWG, and FCR of broilers supplemented with medicinal plants in their diets under HS conditions. A plausible explanation for the increase in growth performance in broiler birds fed medicinal plants could be attributed to the biological compounds present in medicinal plants that improve antioxidant status and the metabolism of protein and fat (37). This experiment also reports that no differences were observed in carcass traits across treatments, except for an increase in spleen percentage in the *Olea Europaea* and a mixture of herbs groups relative to the stressed broiler group. In agreement with our study, Shaker et al. (37) found that feeding broilers with *Artemisia absinthium* leaf powder (1.5%) did not result in significant changes in carcass traits. Chen et al. reported that heat stress could reduce liver function and other immune indices. Certain phytogenic additives work indirectly by deactivating free radicals, which helps to reduce negative effects and maintain a healthy immune system in broilers during heat stress conditions (38, 39). In contrast to our results, Vasilopoulou et al. (40) reported that the addition of 1% olive leaf extract significantly improved meat quality in broilers.

It is known that broiler chickens lack sweat glands, which hinders heat dissipation through evaporation (41). Additionally, their feather-covered bodies restrict heat transfer through conduction, radiation, and convection (42, 43). Therefore, the primary mechanism for heat loss is increased respiratory rate (tachypnea) and shallow breathing (polypnea), resulting in panting, which efficiently removes heat through water evaporation from the respiratory tract (44). Intermittent HS was successfully implemented as indicated by increased rectal temperature and rectal temperature in the experimental treatments (45, 46). Interestingly, chickens treated with medicinal plants showed slightly decreased Tr and RR relative to broiler chickens kept under HS conditions, which is in line with the findings of reference (47). The mechanisms responsible for the antioxidant effects of medicinal plants are not well defined. Our results are consistent with the evidence from reference (4) who mentioned that herbs such as Echinacea, olive, garlic, thyme, and ginger have thermoregulatory action and potential benefits for reducing respiratory rate in poultry due to their robust antioxidant abilities. Generally, these findings are in accordance with those reported by several authors (48–51). Modifications of the phenotypic reactions to medicinal plants during heat stress might be due to different experimental supplementation doses, bioactive compounds, amounts of phenolic and flavonoid compounds, and antioxidant actions.

Cortisol is a stress hormone that plays a crucial role in the physiological response to HS in broiler chickens. When broilers are exposed to high temperatures, their bodies initiate a stress response, which includes the release of cortisol (52). It has been accepted that HS significantly increases serum cortisol levels in broilers compared to the control group. Interestingly, cortisol levels in heat-stressed broiler chickens were not significantly different when compared with the CON group, which is consistent with previous studies (50, 53). It has been reported that exposing broiler chickens to 10 h of HS (32°C) for 7 days did not affect their plasma cortisol levels (54). During the initial phases of HS, plasma cortisol levels in broiler chickens were observed to rise, but they returned to normal levels within 4 days (55). In our study, broiler chickens exposed to heat appeared to have adapted to the elevated temperatures and maintained a regular concentration of plasma corticosterone even after 19 days of heat exposure.

GSH-Px is an antioxidant enzyme typically secreted in the body. A study by reference (56) found that the GSH-Px levels in the HS group were lower than those in the control group. The data support the notion that including phytogenic feed additives with antioxidant properties in diets can help reduce oxidative damage caused by HS in broiler chickens (4, 21, 27, 53). Dissimilarities in heat tolerance among these medicinal plants during heat stress may be due to different secondary metabolites and their mechanisms of action (15, 52).

One aspect affected by heat stress is the immune system, including the synthesis and function of immunoglobulin G (IgG). IgG is a class of antibodies that plays a crucial role in the humoral immune response, providing passive immunity to broilers through maternal transfer and active immunity through their own synthesis. Our results suggest that the supplementation of medicinal plants could improve the release of IgG, especially in broiler-fed diets with *Olea europaea* (51%), relative to broiler chickens in the CON group. In line with our data, a study (57) found improved IgG contents when broiler chickens were supplemented with *Artemisia annua* in HS. Another study by El-Kholy et al. (51) reported that the addition of herbal extracts improved the synthesis of IgG. Additionally, a study by Cheng et al. (50) indicated that Chinese herbs improved immunity in broilers by promoting IgG synthesis in the serum. Furthermore, a study by reference (58) reported that the addition of 1% olive leaf extract significantly improved IgG in broilers. A possible explanation for the positive effects of medicinal plants on IgG may be associated with their role in reducing excessive pro-inflammatory cytokines (IL-8) to maintain a balanced cytokine environment by restoring the integrity of the gut epithelium after HS.

HSP70, a member of the heat shock protein (HSP) family, is recognized as the most widely distributed and evolutionarily conserved family among various organisms (59). An increase in mRNA of *HSP70* is commonly used as an indicator of diverse environmental stress responses, including high temperatures as indicated by reference (60). Growing evidence suggests that the modulation of stressed-induced *HSP70* plays a pivotal role in maintaining a balance between pro-inflammatory and anti-inflammatory cytokines, offering protection against intestinal inflammation (61). HS increased the hepatic mRNA level of HSP70 by 2.7% in stressed broilers relative to the group of thermoneutral broiler chickens (CON). Heat-stressed broiler chickens treated with medicinal plants (*Artemisia sieberi, Achillea fragrantissima*, *Olea europaea*, and their mixture) showed a decrease in hepatic HSP70 mRNA levels by 0.48, 0.37, 0.33, and 0.6%, respectively, compared to stressed broiler chickens.

Similarly, curcumin, a natural polyphenol, inhibited the increase in *HSP70* expression in heat-stressed broilers and quails (56, 62). Additionally, a study by reference (63) found that long-term oral supplementation of flavangenol, derived from pine bark extract, known for its abundant polyphenol content and strong antioxidant properties, led to a reduction in *HSP70* mRNA expression in the livers of broiler chickens experiencing HS. Medicinal plants exhibit multiple mechanisms for their oxidative effects, including radical scavenging abilities and synergy with other antioxidants, as discussed by previous research (64).

Occludin, an integral protein within the tight junctions of intestinal cells, plays a crucial role in creating selective barriers that regulate paracellular transport (57). The impairment of tight junctions results in an increase in the paracellular permeability of intestinal contents, which is recognized as a hallmark of various pathological conditions (65).

HS decreased ileum mRNA abundance of *OCLN* in stressed broiler groups (T1) relative to CON broiler chickens. A previous study (57) observed a decrease in the gene expression of occludin proteins in the jejunal mucosa of heat-stressed broilers. Similarly, broilers exposed to cyclic HS showed a decrease in mRNA abundance of *OCLN* in the jejunal mucosa at 21 days and a decrease in mRNA abundance of OCLN in both the jejunal and ileal mucosa at 42 days. These findings, coupled with compromised intestinal morphology, suggest that cyclic HS can disrupt the integrity of the intestinal barrier (64, 65). Conversely, heat-stressed broiler chickens treated with the medicinal plants used in this study, especially *Olea europaea*, and their mixture of investigated medicinal plants exhibited an increase in ileum mRNA abundance of *OCLN* compared to stressed broiler chickens.

One possible explanation for this phenomenon could be that the increase in this protein indicates a barrier enhancement effect during HS (52) as a compensatory mechanism for increased permeability. This idea is supported by data showing that heat-induced expression of HSPs is necessary for the expression of OCLN (57, 66). Previous authors found that HS reduced the protein levels of OCLN in the jejunum compared to the thermoneutral zone (57, 66). Furthermore, Artemisia supplementation significantly increased ileal OCLN mRNA expression levels in response to HS, suggesting that Artemisia could improve intestinal barrier function (57). A study by reference (67) demonstrated that heat exposure significantly increased the expression of mRNA of pro-inflammatory cytokines such as IL-8 in the chicken ileum as indicated in the current research. HS increased the mRNA abundance of IL-8 in stressed broilers compared to control broiler chickens. As expected, broiler chickens treated with medicinal plants (*Artemisia sieberi* and a mixture of herbs) showed decreased mRNA abundance of *IL-8* compared to stressed broiler chickens. These results may indicate the anti-inflammatory effects of herbs used to alleviate the negative effects of HS in various animals as reported by several authors (7, 68, 69). Numerous studies have provided evidence that HS can lead to intestinal dysfunction (70, 71). HS has been observed to cause a decline in the quality of intestinal morphology, characterized by impaired villus-crypt architecture, reduced villus height, and diminished villus surface area (71). These alterations in intestinal structure, such as shorter villi and deeper crypts, have been associated with compromised nutrient absorption, increased electrolyte and water secretion in the gastrointestinal tract, and subsequently reduced performance (57). Supplementation with medicinal plants or herbs has shown improvement in certain morphological aspects of intestinal tissues. Specifically, treatment with medicinal plants has been associated with increased villus height, crypt depth, and villus surface area (72, 73). The enhanced gut integrity observed with medicinal plant supplementation may be attributed to their role in promoting intestinal cell proliferation (74).

## 5 Conclusion

Supplementing broiler chickens with dietary medicinal plants during HS challenges may enhance their antioxidant status and anti-inflammatory responses. The improved heat tolerance in broiler chickens treated with medicinal plants leads to an increase in antioxidant potential in heat-stressed broiler chickens. We observed improvements in GSH-Px and IgG levels, a decrease in hepatic HSP70 mRNA levels, an increase in ileum mRNA *OCLN* levels, and a decrease in *IL-8* mRNA levels. These responses contribute to enhancing the gastrointestinal tract by increasing villus height and surface area while decreasing crypt depth.

## Data availability statement

The original contributions presented in the study are included in the article/supplementary material, further inquiries can be directed to the corresponding author.

## Ethics statement

The animal study was approved by the Animals Ethics Committee of the deanship of scientific research at the University of Jordan (Amman, Jordan). The study was conducted in accordance with the local legislation and institutional requirements.

## Author contributions

ZM: Conceptualization, Data curation, Formal analysis, Funding acquisition, Investigation, Methodology, Project administration, Resources, Software, Supervision, Validation, Visualization, Writing – original draft, Writing – review & editing. MA: Conceptualization, Data curation, Formal analysis, Funding acquisition, Investigation, Methodology, Project administration, Resources, Software, Supervision, Validation, Visualization, Writing – original draft, Writing – review & editing. AA: Writing – original draft, Writing – review & editing. MA-Q: Writing – original draft, Writing – review & editing. MA-M: Writing – original draft, Writing – review & editing. HA-T: Writing – original draft, Writing – review & editing. HZ: Writing – original draft, Writing – review & editing. AA-F: Conceptualization, Data curation, Formal analysis, Funding acquisition, Investigation, Methodology, Project administration, Resources, Software, Supervision, Validation, Visualization, Writing – original draft, Writing – review & editing.
